# Supplementary material for: Quasiadiabatic electron transport in room temperature nanoelectronic devices induced by hot-phonon bottleneck
Source: Nat Commun. 2021 Aug 6;12:4752. doi: 10.1038/s41467-021-25094-5 (PMC8346506; doi:10.1038/s41467-021-25094-5)
Supplement: Supplementary file 1 — Supplementary Information [file 41467_2021_25094_MOESM1_ESM.pdf]

Supplementary Information

Quasiadiabatic electron transport in room temperature nanoelectronic devices induced by hot-phonon bottleneck

Qianchun Weng[1,2,§,#,*], Le Yang[3,#], Zhenghua An[3,4,#,*], Pingping Chen[1], Alexander Tzalenchuk[5,6], Wei Lu[1,7,*] & Susumu Komiyama[1,8,9]

[1] National Laboratory for Infrared Physics, Shanghai Institute of Technical Physics, the Chinese Academy of Sciences, Shanghai 200083, China.

[2] Institute of Industrial Science, The University of Tokyo, Komaba 4-6-1, Meguro-ku, Tokyo 153-8505, Japan.

[3] State Key Laboratory of Surface Physics, Institute for Nanoelectronic Devices and Quantum Computing, and Department of Physics, Fudan University, Shanghai 200433, China.

[4] Shanghai Qi Zhi Institute，41th Floor, AI Tower, No. 701 Yunjin Road, Xuhui District, Shanghai, 200232, China.

[5] National Physical Laboratory, Hampton Road, Teddington, TW11 0LW, United Kingdom.

[6] Royal Holloway, University of London, Egham TW20 0EX, United Kingdom.

[7] School of Physical Science and Technology, ShanghaiTech University, Shanghai, 201210, China.

[8] Department of Basic Science, The University of Tokyo, Komaba 3-8-1, Meguro-ku, Tokyo 153-8902, Japan.

[9] Terahertz Technology Research Center, National Institute of Information and Communications Technology, Nukui-Kitamachi 4-2-1, Koganei, Tokyo 184-8795, Japan.

[§] Present address: Surface and Interface Science Laboratory, RIKEN, 2-1 Hirosawa, Wako, Saitama 351-0198, Japan.

[#] These authors contributed equally to this work.

[*] Corresponding authors: Z.A. (anzhenghua@fudan.edu.cn), Q.W.(qcweng@gmail.com) and W.L. (luwei@mail.sitp.ac.cn)

Supplementary Notes

Note 1. What is measured with SNoiM

Note 2. Scanning thermal microscope (SThM)

Note 3. Experimental profiles of T_e and T_L

Note 4. Effective temperature of LO-phonons: T_{LO}

Note 5. Estimation of E in the channel

Note 6. Self-consistent determination of T_{LO} in a simplified model

Note 7. Intervalley transfer of electrons in GaAs

Note 8. Theoretical estimation of T_e and P_{LO} in two-carrier transport

Note 9. Broadening of T_L distribution

Supplementary Figures

Fig. 1. EM-LDOS for the GaAs/AlGaAs QW structure used in this work.

Fig. 2. Decay profile of the near-field (NF) signal.

Fig. 3. Images of T_e and T_L obtained for opposite bias polarities.

Fig. 4. 2D and 1D image of T_L for V_b = +8.0 V.

Fig. 5. Energy profile of the conduction band of GaAs.

Fig. 6. Simulated electric field distribution.

Fig. 7. T_{LO} vs. p curve and p_{LO} vs. T_{LO}.

Fig. 8. Expected values of important physical quantities along the channel of the device.

Fig. 9. Suppression ratio of energy loss due to the hot-phonon bottleneck effect.

Supplementary Note 1 What is measured by Scanning Noise Microscope (SNoiM)

SNoiM is currently the only experimental tool available to probe electron temperature T_e at the nanoscale. The configuration of the equipment is similar to the passive scattering-type scanning near-field optical microscope (s-SNOM) utilizing a ultrahigh sensitivity detector CSIP.[1,2] The technique has been applied to successfully image fluctuating electro-magnetic evanescent fields on (i) metals and dielectrics in thermal equilibrium,[3-5] (ii) Joule-heated metal wires[6] and (iii) current-driven hot electrons in semiconductor nanostructures.[7] Detailed discussion on what is measured with SNoiM is given in Ref. 8 and in Supplementary Materials of Ref. 7, including experimental confirmation of derived formulae as well as the experimental procedure of deriving T_e. Below is a brief summary of the discussion.

Experimentally, it is essential that SNoiM detects only the evanescent fields localized on the material surface within a distance less than 1% of the target wavelength λ, and is perfectly insensitive to or unaffected by the radiation in the far field (at a distance > 0.01 λ). This is assured by modulating the probe-tip height and taking modulation signals.[5,7,8] This is the reason why SNoiM is insensitive to the familiar blackbody radiation,[9] the THz photon emission induced by coherently driven electron motion,[10] and the photons emitted via one-particle radiative transition.[11] Detected with SNoiM is the tip-scattered fluctuating evanescent waves, the intensity of which is proportional to the local energy density, $u(z, \omega)$, of the electro-magnetic (EM) field at the position z of the probe tip. In this work ($\lambda = 14.5 \pm 0.8$ μm), the detected angular frequency is $\omega = 2\pi\nu = 2\pi(c/\lambda) = 130 \pm 7.5$ THz. (The spatial resolution of SNoM is primarily determined by the probe tip radius, typically 30~60 nm.[12]) This ability of SNoiM, sensing the EM energy density, is the distinguished feature absent in the conventional near-field optical microscope, which probes complex dielectric constants but not directly the energy density.[13] If a half-infinite ($z<0$) material of plane interface is in thermal equilibrium at temperature T_s, theory shows that thermally-agitated current/charge fluctuation in the material generates the EM energy density,[14,15]

$$u(z, \omega, T_s) = \rho(z,\omega)[\hbar\omega/\{\exp(\hbar\omega/k_B T_s) - 1\}], \qquad \text{(S1)}$$

at distance $z>0$ from the interface, where $\hbar$ is the Dirac constant and k_B is the Boltzmann constant. $\rho(z,\omega)$ is the material-specific EM local density of states (EM-LDOS), which is given in terms of

the complex dielectric constants of the material: In general, ρ (z,ω) decreases rapidly with increasing z as will be shown in Supplementary Fig. 1(b) and Supplementary Fig. 2, manifesting the feature of evanescent waves. Physically, Eq.(S1) is regarded as a generalized expression of the Johnson–Nyquist noise expanded to the evanescent waves on all the materials including dielectrics. Joulain and his collaborators [Sec. IV of Supplementary Ref.16] showed theoretically that Eq.(S1) is tied to what is detected in the far fields when thermally excited evanescent fields are scattered with a tip (i.e. by SNoiM) in the thermal equilibrium condition. Experimentally, a series of measurements of SNoiM on metals and dielectrics in the thermal equilibrium condition have been carried out to support Eq.(S1).

When conduction electrons are away from equilibrium with the lattice, the two subsystems have to be treated separately. If the electron energy distribution function and the phonon distribution function can be charactered, respectively, by the effective electron temperature T_e and the effective lattice temperature T_L, Eq.(S1) is replaced with the generalized expression

$$u\,(z,\omega,T_e,T_L) = \rho_e\,(z,\omega)[\hbar\omega/\{\exp(\hbar\omega/k_B T_e) - 1\}] + \rho_L(z,\omega)[\hbar\omega/\{\exp(\hbar\omega/k_B T_L) - 1\}], \qquad \text{(S2)}$$

which comprises separate contributions from the conduction electrons and the lattice, respectively characterized by the EM-LDOSs, ρ_e and ρ_L, and the effective temperatures, T_e and T_L. Equation (S2) makes remarkable approximation to characterize the highly non-equilibrium electrons by just one parameter T_e. This is justified by the fact that the energy exchange within the electron system is frequent enough to approximately establish T_e. This is assured in the present study because the electron-electron scattering rate, $1/\tau_{ee}$~1/(40fs), is much higher than the electron-phonon energy relaxation rate, $1/\tau_{e\text{-}ph}$ ~1/(1.2 ps) even in the highest bias conditions. The justification here does not necessarily imply that the Fermi function in the thermal equilibrium at $T = T_e$ closely resembles the true electron distribution function. Instead, the key is that, despite possible dissimilarities, any physical quantities obtained by assuming T_e give reasonable approximation to the true values. [Strictly, characterizing the lattice system by just one parameter, T_L, is not safely justified, but the discussion in this work is unaffected because the contribution from the lattice system is negligibly small in our SNoiM measurements as described below and as experimentally confirmed in this work.] Supplementary Figure 1 (a) shows theoretically derived values of ρ_e , ρ_L and $\rho_e + \rho_L$ of the

GaAs/AlGaAs QW structure used in this work. Since $\rho_L << \rho_e$ at $\lambda \approx 14.5$ μm (Supplementary Fig. 1 (a)) and $T_L << T_e$ (V_b >3.0V) in this work, the energy density u is dominated by the contribution from electrons while the lattice contribution is negligibly small (less than 0.3%). The signal of SNoiM is hence regarded as the hot-electron shot-noise. Supplementary Figure 1(b) shows that the electron-induced p-polarized electric-field component, $\rho_e^{p\text{-}E}$ (red solid line), dominates the evanescent field (or u) and that it rapidly decreases with increasing z.

Fundamental validity of the theoretical framework described in the above has been confirmed in a series of experiments on the material dependence, the temperature dependence and the z-dependence of the SNoiM signal. Particularly, crucial test is made through the quantitative analysis of the rapid decay profile of evanescent field with increasing z, confirming definite agreement between the theory and experiments.[4,5,7,8,12] Supplementary Figure 2 displays an example of decay profiles of the SNoiM signal taken at the hot spot near the entrance and the one near the exit in the device of Figs. 1 and 2 at V_b = 8.0 V. Both of the hot spots are visible only in the region very close to the sample surface (z < 100 nm ≈ 0.007xλ), consistent with the theoretical prediction of Supplementary Fig. 1(b).

In this work, both of the electron system and the phonon system are, respectively, in nonequilibrium states. For considering the signal of SNoiM, nonequilibrium phonon distribution can be ignored because the total hot-phonon energy is small and its possible contribution to SNoiM signal is negligibly small regardless of the fashion how the phonons are distributed. As to the electron system, mutually nonequilibrium Γ-valley electrons and X-valley electrons are taken into account in the analysis by introducing the mean electron temperature given by Eq. (3):

$$< T_e >= \frac{n_\Gamma}{n} \cdot T_\Gamma + \frac{n_X}{n} \cdot T_X,$$

where n_Γ, n_X, T_Γ and T_X are the fractional densities and the effective temperatures of the electrons in respective valleys. This is a simplified approximation because (i) the EM-LDOS in Eq. S2, ρ_e, is approximately used as a common parameter, (ii) the electron energy distribution function, in either valley, will not be accurately described by the Fermi function labeled by T_Γ or T_X, and (iii) the intervalley transition of electrons yields additional contribution to the shot noise. Nevertheless, these are justified approximations, and the discussion and the interpretation in this work are valid.

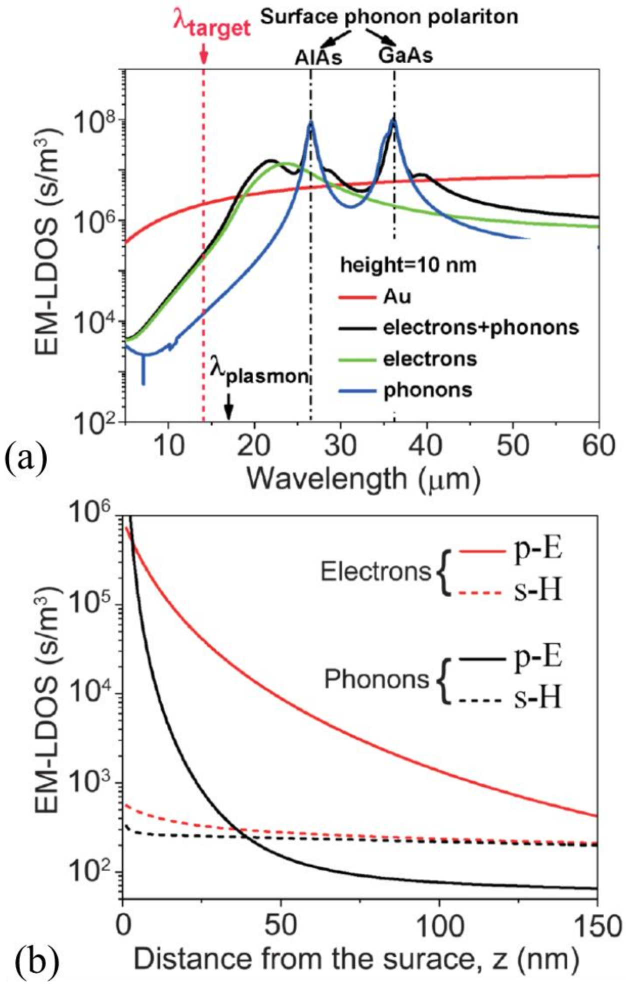

Supplementary Figure 1. Electromagnetic local density of states (EM-LDOS) for the GaAs/AlGaAs QW structure used in this work. (a) Theoretically derived values at z=10 nm as a function of the wavelength. The total EM-LDOS (black line) is a sum of the contributions from the electrons ρ_e (green line) and the phonons ρ_L (blue line). Reference data of gold, ρ_{Au} (red line), is shown together. Two dominant peaks in ρ_L are due to the surface phonon polariton resonances of GaAs and AlAs, respectively. At the target wavelength is λ_{target}= 14.5 μm, the contribution of conduction electrons ρ_e (green line) dominates over that of phonons ρ_L (blue line). (b) Dependence on the distance z of ρ_e and ρ_L, where each contribution comprises the p-polarized component and the s-polarized component, $\rho_e = \rho_e^{p\text{-}E} + \rho_e^{s\text{-}H}$ (electrons) and $\rho_L = \rho_L^{p\text{-}E} + \rho_L^{s\text{-}H}$ (phonons). In the actual measurement of imaging (z = 20 ~ 50 nm), $\rho_e^{p\text{-}E}$ dominates which rapidly decreases with increasing z. (Replot of Supplementary Fig. 7 in Supplementary Materials of Ref. 7.)

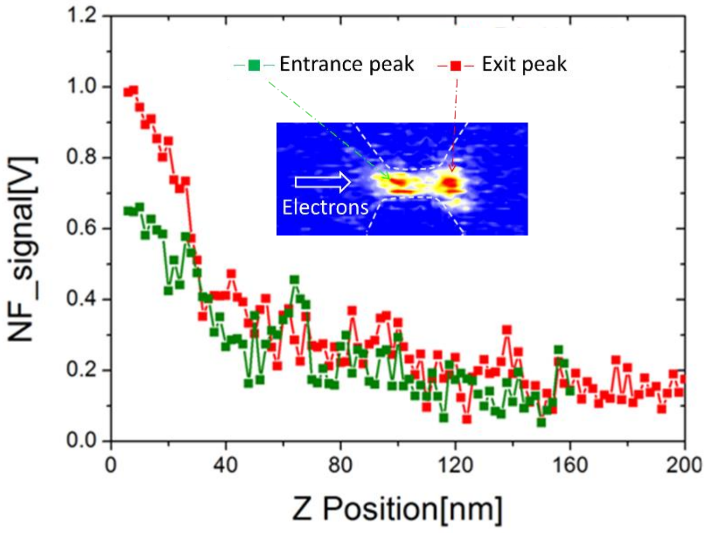

Supplementary Figure 2. Decay profile of the near-field (NF) signal studied at the hot spots near the entrance and the exit in the device of Figs.1 and 2 at V_b = 8.0V.

Supplementary Note 2 Scanning thermometry Microscope (SThM)

The spatial resolution of the equipment used in the present study (ANASYS INSTRUMENTS, NanoTA) is nominally 20 nm. The realistic resolution is supposed to be ~50 nm in the present experiment made in the ambient condition, where the tip-sample contact is dominated by the liquid bridging.[17] We noticed that edge-related artifact signals occasionally show up, similarly to the report in Ref. 18. The artifact signals, however, are relatively small depending crucially on the probe tip used, the surface condition, the fine location of the scanned trajectory and the mesa-etch depth. In all the measurements presented in this work, we have avoided the artifact signals by paying careful attention to the choice of the probe tip, fine trajectories of scan and the preparation of the sample-surface condition.

Aside from the technique applied in this work utilizing the thermocouple- or the thermistor-integrated AFM tip, several different techniques have been developed.[18,19] Examples include fluorescent microscopy [20], Joule-expansion microscopy [21], superconducting quantum interference device (SQUID)[22,23], thermal expansion induced Plasma frequency shift [24], and surface-intensified Raman spectroscopy[25]. It should be mentioned that in all those techniques, probed is the lattice temperature or the phonon temperature of particular modes, not the electron temperature. Potentially, the scanning microscope of near-field heat transfer [26] is supposed to detect the electron temperature but its capability has not been demonstrated experimentally.

Supplementary Note 3 Experimental profiles of T_e and T_L

Absolute values of T_e in Figs. 1-3 are derived by normalizing the signal intensity (bias voltage V_b), $I_{GaAs,Vb}$, with the experimental value of Au in thermal equilibrium at T_{Room} = 300 K, $I_{Au,0}$, through the equation $I_{GaAs,Vb} / I_{Au,0} = (\rho_e / \rho_{Au}) \times \{\exp(\hbar\omega/k_B T_{Room}) - 1\} / \{\exp(\hbar\omega/k_B T_e) - 1\}$ derived from Eq. S2 by ignoring the phonon contribution. Since ρ_e / ρ_{Au} is theoretically known to be 10.0 in Supplementary Fig. 1 (a), T_e is derived without using any adjustable parameter.

Additional experiments on devices similar to those of Figs. 1 and 2 with channel lengths ranging from 200 nm to 1.0 μm, have been carried out to confirm the systematic difference between the T_e- and the T_L-profiles as described in the text. Supplementary Figure 3 displays images of T_e and T_L in the opposite polarities of V_b, obtained on the device of Fig. 1.

Differently from the T_e-distribution in Supplementary Fig. 3a, the T_L-distribution in Supplementary Fig. 3b is, approximately, circularly symmetric about the shifted center. If finite energy is dissipated to the lattice in the channel, it would lead to an enhanced tail of elevated T_L developing towards the channel entrance. One-dimensional profiles of T_L displayed in Supplementary Fig. 3c for the opposite bias polarities show that systematic asymmetry is not discerned, so that finite energy loss in the channel is indicated to be insignificant.

The lattice temperature of the sample substrate in the larger region outside the imaged area, $(x^2+y^2)^{1/2} > 5$ μm, is noted to be nearly uniform at T_L = 300.8 K for V_b = ±8.0 V, which is higher by 0.6 K than the ambient temperature or the sample temperature $T_e = T_L$ = 300.2 K obtained without bias (V_b = 0). This is because the sample substrate is slightly heated up uniformly due to the contact thermal resistance between the backside surface of sample substrate and the sample stage. Hence, we define $T_L = T_{base}$ = 300.8 K to be the background base temperature ($\Delta T_L = 0$) for V_b = ±8.0 V, as marked with the dash-dotted line in Supplementary Fig. 3c. (The baseline is taken similarly in Fig. 1b, and Fig. 3 a & d.) Supplementary Figures 4 a-c elucidate that T_L distribution is broadened similarly in the y-direction normal to the channel.

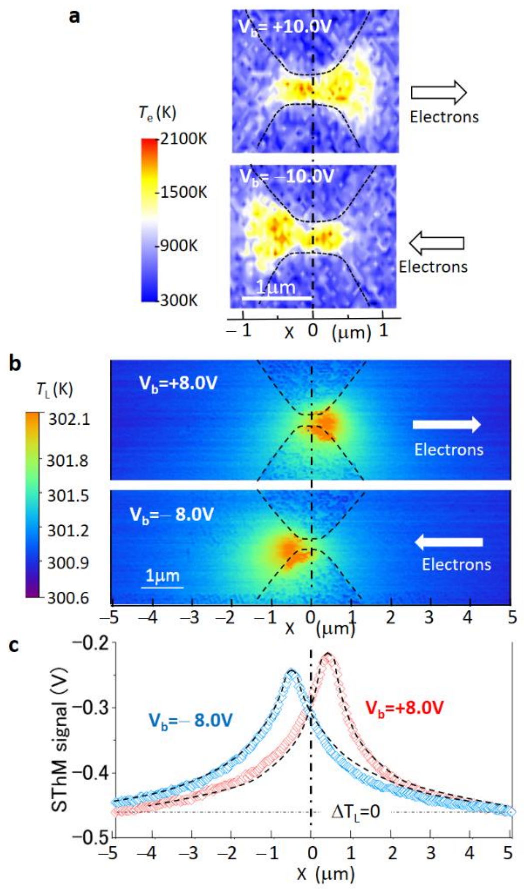

Supplementary Figure 3. Distributions of T_e and T_L obtained, respectively, with SNoiM and SThM for opposite bias polarities. 2D images of (a) T_e for $V_b = \pm 10.0$ V and (b) T_L for $V_b = \pm 8.0$ V. (c) 1D profiles of T_L along the channel in the x-direction (y = 0), where the horizontal line ($\Delta T_L = 0$) marks the background lattice temperature of the sample substrate ($T_L = 300.80$K). Broken lines are drawn to show symmetric curves for respective polarities. Systematic asymmetry is not discerned in the experimental curves.

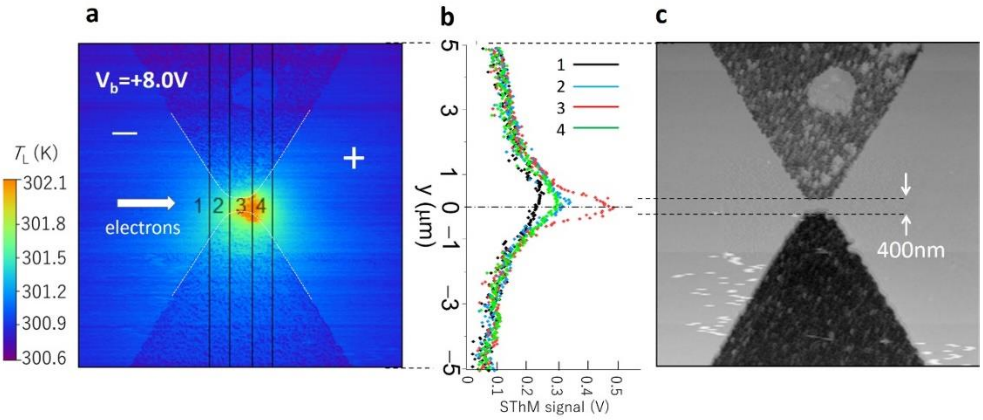

Supplementary Figure 4. (a) 2D image of T_L for $V_b = +8.0$ V on the same device as for Supplementary Fig. 3. (b) 1D-scan of T_L along the lines 1~ 4 crossing the channel in the y-direction. (c) Topographic image of the device taken with an atomic force microscope.

Supplementary Note 4 Effective temperature of LO-phonons: T_{LO}

Hot electrons in the Γ-valley is primarily confined in a small k-space sphere around the zone center with radius $k_\Gamma = 1.0\times10^9$ m^{-1} given by $(\hbar k_\Gamma)^2/(2m^*_\Gamma) = \Delta\varepsilon_{\Gamma X}$ with $m^*_\Gamma = 0.067\ m_0$ as indicated with a circle in Supplementary Fig. 5 a. This is because $\Delta\varepsilon_{\Gamma X} = 550$ meV >> $k_B T_e$ ($T_e \approx 2000$ K) and $\Delta\varepsilon_{\Gamma X}$>> E_F in the present experimental condition. The wave number q of an LO-phonon emitted by a Γ-valley electron with wave number k is smaller than 2k in general. Hence, we assume that the LO phonons emitted by Γ-valley electrons are similarly confined in a small sphere with radius q = $k_\Gamma = 1.0\times10^9$ m^{-1}. The number of LO-phonon states accommodated in the sphere of q = k_Γ, per unit area of the d = 35 nm-thick QW, is NOS $=\{k_\Gamma/(2\pi)\}^3 d \approx 1.4\times10^{17}$m^{-2}.

The emitted LO phonons stay within this limited small q-space volume for the decay lifetime of about 5.0 ps. The LO-phonon occupation number, $n_{LO}= N_{LO}/NOS$, is derived from the number of emitted phonons N_{LO}, from which T_{LO} is derived through $n_{LO}= \{\exp(\hbar\omega_{LO}/k_B T_{LO})-1\}^{-1}$. The high values of T_{LO} in the present work arise from (i) the high rate of LO-phonon emission, (ii) the small q vectors of emitted LO-phonons, and (iii) the real-space confinement in a quantum well (small d).[27]

The LO-phonons absorbed/emitted by electrons within each X-valley are also limited in a small q-space sphere around the zone center: Although the sphere is slightly larger than that for the Γ valley, the difference is insignificant for the present analysis.

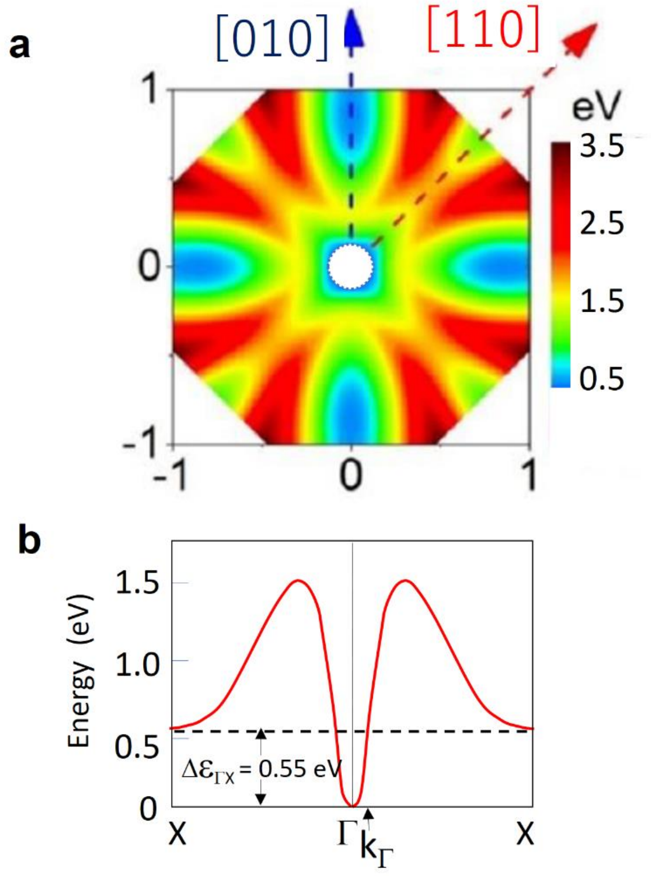

Supplementary Figure 5. Energy profile of the conduction band of GaAs. The bottom of the conduction band is set to be $\varepsilon = 0$. (a) 2D color plot on the (001) plane. The white circle area around the center indicates the region of $k<k_\Gamma$. (b) 1D profile along the <100> direction.

Supplementary Note 5 **Estimation of *E* in the channel**

The electric field distribution $E(\mathrm{x})$ for the device geometry of Figs. 1 and 3 is derived for $V_b = 8$ V via simulation calculation based on the finite-element method. As the first step of simulation, the solid black line in Supplementary Fig. 6 is obtained first by assuming linear transport. The curve shows intensification of $E(\mathrm{x})$ due to geometrical effect of the constriction channel. The red broken line is, in turn, obtained by additionally taking into account the non-linear transport effect; viz., the local resistivity increase induced by $E(\mathrm{x})$. (The resistance increases by a factor up to 5 at the channel center.) The red broken line gives the physically realistic profile of $E(\mathrm{x})$ for $V_b = 8$ V, which exhibits stronger concentration into the channel region reaching ~ 40 kV/cm, consistent with the experimentally estimated value ~ 40 kV/cm from the current voltage characteristics (the bottom of Fig. 1c). We mention that the solid black line is similar to the profile of $E(\mathrm{x})$ for $V_b = 3$ V, when the vertical scale is modified so that the peak value corresponds to ~ 4.5 kV/cm.

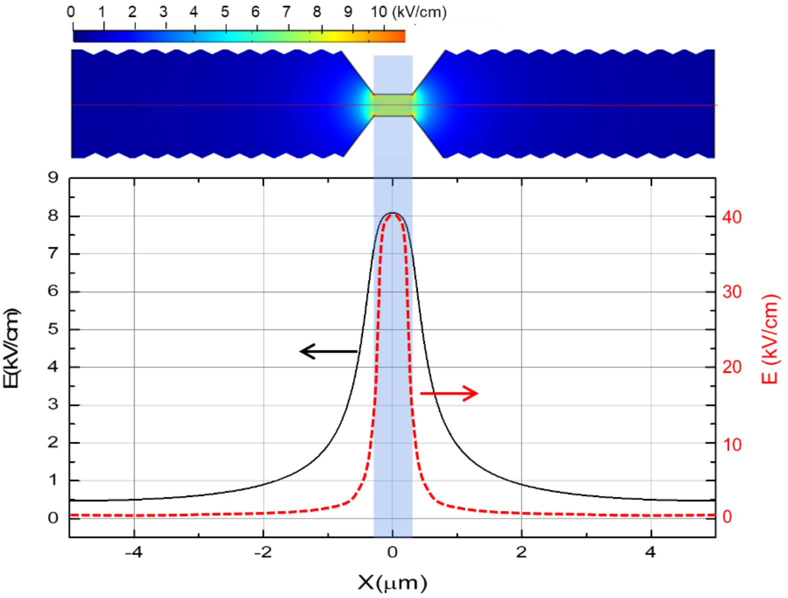

Supplementary Figure 6. Simulated electric field distribution of the device used for the data of Fig.1 at V_b=8.0V (red broken line). The solid black line is similar to the profile of E(x) for V_b = 3 V, when the vertical scale is modified so that the peak value corresponds to ~ 4.5 kV/cm.

Supplementary Note 6 Self-consistent determination of T_{LO} in a simplified model

In the hot-phonon bottleneck regime, the value of T_{LO} estimated from Eq. (1) is not realized because the electron system absorbs energy from the phonon system to lower T_{LO}. The physically realized value of T_{LO} is estimated from the relation

$$k_B T_{LO} = \hbar\omega_{LO}/\ln[1+\{AP_{LO} + n_{LO}(T_{Room})\}^{-1}], \quad \text{(S1)}$$

where $p = E\,j$ in Eq. (1) is replaced with P_{LO}. Here P_{LO} is the net energy dissipation of hot electrons to the LO-phonon system, which takes account of both the emission and the absorption of LO phonons. The blue line in Supplementary Fig. 7 shows the values of T_{LO} against P_{LO} according to Eq.(S1). When two types of carries (in Γ- and X-valleys) are taken into account, $P_{LO} = P_\Gamma + P_X$ given by Eq. (4) in the text has to be taken. However, we apply here a simplified model of single-carrier transport to make possible straightforward determination of T_{LO}. Hence P_{LO} is written as

$$P_{LO}(T_{LO}, T_e) = \frac{\Delta\varepsilon}{\tau_{LO} D_0} \int_0^\infty d\varepsilon\, D(\varepsilon) D(\varepsilon + \Delta\varepsilon)$$
$$\times [f(\varepsilon + \Delta\varepsilon) \cdot \{1 - f(\varepsilon)\} \cdot (n_{LO} + 1) - f(\varepsilon) \cdot \{1 - f(\varepsilon + \Delta\varepsilon)\} \cdot n_{LO}], \quad \text{(S2)}$$

where the energy distribution of electrons is approximated by the Fermi function

$$f(\varepsilon) = 1/[\exp\{(\varepsilon - \mu)/k_B T_e\} + 1]$$

characterized by the single parameter T_e with μ determined by $n = \int_0^\infty f(\varepsilon) D(\varepsilon) d\varepsilon$. The density of states of the electrons is given by $D(\varepsilon) = D_\Gamma(\varepsilon)$ for $\varepsilon < \Delta\varepsilon_{\Gamma X}$ and $D(\varepsilon) = D_\Gamma(\varepsilon) + D_X(\varepsilon)$ for $\Delta\varepsilon_{\Gamma X} < \varepsilon$. The LO-phonon distribution is given by T_{LO} through

$$n_{LO} = \{\exp(\hbar\omega_{LO}/k_B T_{LO}) - 1\}^{-1}.$$

The quantities, $D_\Gamma(\varepsilon)$, $D_X(\varepsilon)$, $\Delta\varepsilon = \hbar\omega_{LO}$, $\Delta\varepsilon_{\Gamma X}$, τ_{LO} and D_0, are the same as those given for Eq. (4).

When T_e is known at a given electrical input power $p = Ej$, one can determine T_{LO} by combining Eqs. (S1) and (S2). In the high bias condition of Fig. 3a (p = 7.72x10^{10} W/cm^3, V_b = 8.0V), T_e = 2,000 K is experimentally found. The values of P_{LO} given by Eq.(S2) with T_e = 2,000 K are shown against T_{LO} with the red line in Supplementary Fig. 7. The fact that T_{LO}=7,300 K is derived from Eq. (1) (Fig. 4) implies that T_{LO}=7,300 K would be expected if P_{LO} = p= 7.72x10^{10} W/cm^3 in Supplementary Fig. 7, as marked by the blue triangle. The physically realized value of T_{LO} is determined by the intersection of the two curves of Eqs. (S1) and (S2) in Supplementary Fig.

7 marked by the double cross circle; viz., T_{LO} = 1,990 K at p_{LO} =1.83x10^{10} W/cm^3. Thus the value of T_{LO} is close to but slightly lower than T_e = 2,000 K. The energy dissipation to the LO-phonon system is accordingly suppressed to a small fraction, P_{LO}/p = 0.235, of the electrical input power p. As schematically illustrated with a gray arrow in Supplementary Fig. 7, the suppression ratio of the hot-phonon bottleneck effect (for a given T_e) is defined by

$$\gamma_{supp} = P_{LO}(T_{LO})/P_0, \qquad \text{(S3)}$$

where $P_{LO}(T_{LO})$ is the realized true energy dissipation (crossed circle in Supplementary Fig. 7) and $P_0 = P_{LO}(T_{Room})$ is the fictitious value of dissipation without the hot-phonon effect (white circle in Supplementary Fig. 7), derived from Eq.(S2) by setting T_{LO}=T_{Room} and T_e=2,000 K. The suppression ratio is $\gamma_{supp} = P_{LO}(T_{LO})/P_0$ = 0.37% for T_e=2,000 K at p= 7.72x10^{10} W/cm^3 (V_b = 8.0 V), where $P_{LO}(T_{LO})$ = 1.83x10^{10} W/cm^3 and $P_{LO}(T_{Room})$ = 5.0x10^{12} W/cm^3 with T_{Room}=300 K.

As shown in the above, the existence of hot-phonon bottleneck effect and its significant impact on the energy dissipation in the hot-electron transport are definitely supported in the simplified single-carrier model. (In the simplified model, the reduction of electron energy distribution at higher energy levels may be somewhat underestimated and the hot-phonon bottleneck effect may be slightly overestimated in quantitative terms. Nevertheless, the concept of discussion is justified yielding semi-quantitatively valid results.)

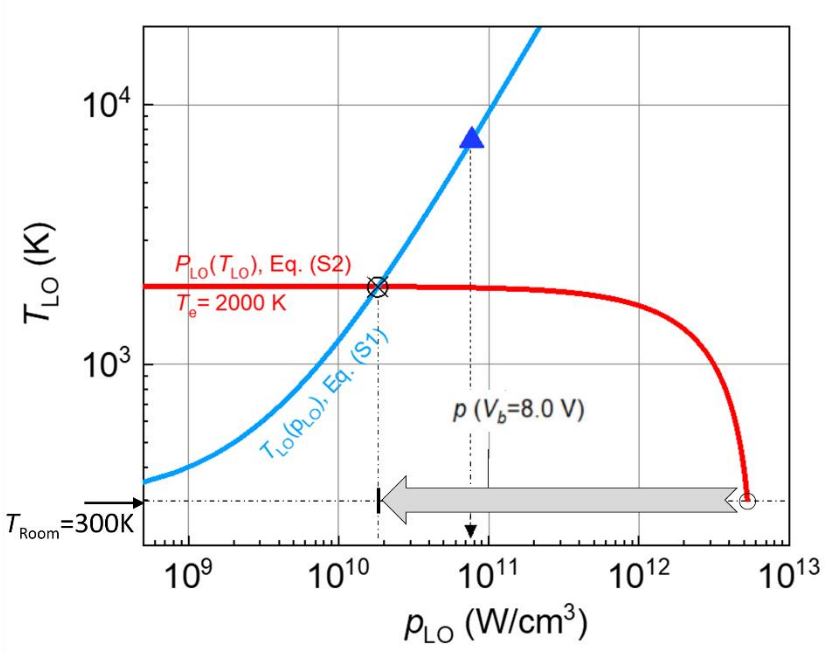

Supplementary Figure 7**.** Self-consistent determination of T_{LO} realized when T_e = 2,000K at p = 7.72x10^{10} W/cm^3 (V_b=8.0V). The blue triangle marks T_{LO} = 7,300K, which would be realized if $P_{LO} = p$ = 7.72x10^{10} W/cm^3. The blue line shows the T_{LO} versus P_{LO} curve given by Eq.(S1). The red line shows the P_{LO} versus T_{LO} curve for T_e=2,000K according to Eq.(S2). The intersection of the two curves (marked by the double cross circle ⊗) gives the physically realized values of T_{LO} and P_{LO}; viz., T_{LO} = 1,990K at P_{LO} =1.83x10^{10}W/cm^3. The limiting value of Eq.(S2), $P_{LO}(T_{Room})$, at $T_{LO} = T_{Room}$=300K is P_{LO} = 5.0x10^{12}W/cm^3 as marked by a white circle. The gray arrow schematically illustrates the suppression of energy dissipation due to the hot-phonon bottleneck effect.

Supplementary Note 7 Intervalley transfer of electrons in GaAs

Detailed description of the intervalley transfer can be found in Refs. 28-31 and in Ref. 32 and Supplementary Materials of Ref. 7. Brief description is given below. Hot electrons in the Γvalley begin to transfer into X valleys as E exceeds ~ 10 kV/cm. The effective mass tensor of electrons in X valleys is anisotropic, described by $(m_X^{//}, m_X^{\perp}, m_X^{\perp}) = (1.98m_0, 0.27m_0, 0.27m_0)$ with a large density-of-state effective mass $m^X_d = (g^2 m_X^{//} m_X^{\perp} m_X^{\perp})^{1/3} = 1.09m_0$, where $g = 3$ is the number of equivalent valleys.[32,33] The Γ→X intervalley scattering time is typically $\tau_{\Gamma\rightarrow X} \approx 45$ fs.[32] While each event of intervalley transfer is accompanied by emission or absorption of zone-boundary LO-phonons, the relevant energy gain and loss are ignored in our analysis because they nearly compete to balance each other in the present experimental condition.

Intervalley transfer to upper satellite L valleys (valley splitting energy $\Delta\varepsilon_{\Gamma L} \approx 250$ meV) takes place as well, causing Gunn effect. The rate of the Γ→L intervalley scattering is, however, relatively slow, typically $1/\tau_{\Gamma\rightarrow L} \approx 1/450$ fs.[32] It is hence important in relatively long channels (> 1 μm) at moderately intense electric fields ($E \approx 1.5$ ~ 10 kV/cm), but insignificant in short channels (< 1 μm) at higher electric fields ($E > 10$ kV/cm).[32] Accordingly the L-valley transfer can be ignored in the present work.

Supplementary Note 8 Theoretical estimation of T_e and P_{LO} in two-carrier transport

Nonequilibrium distribution between electrons and phonons in GaAs short channel conductors have been treated via simulation calculation,[34] where hot-electron temperature exceeding 1000 K is reported. However, the rise in the LO-phonon temperature is estimated to be as small as $\Delta T_{LO} \approx 20$ K. This is because the analysis derives ΔT_{LO} by averaging over the entire Brillouin zone, whereas the emitted LO-phonons are in fact confined in a limited narrow q-space volume as discussed already (Supplementary Fig. 5).

Described below is the procedure of estimating the profiles of T_e and T_L shown in Figs. 3c and d. The device shown in Fig. 1 and Supplementary Fig. 3 (V_b = 8.0 V) is considered by referring to Monte Carlo simulation,[35] in which fractional electron densities, n_Γ, n_L, n_X, and the average electron kinetic energies, $k_B T_\Gamma$, $k_B T_L$, $k_B T_X$, for respective valleys are calculated against E. In these calculations of Ref. 35, long conduction channels are implicit and phonons are assumed to be in thermal equilibrium with the environment (room temperature). For E = 5, 10, 20, 30, 40 kV/cm, (n_Γ/n,n_L/n, n_X/n) varies as (0.42,0.55, 0.03), (0.19, 0.67, 0.14), (0.22,0.41, 0.37), (0.21, 0.27, 0.52), (0.20,0.20, 0.60) and ($k_b T_\Gamma$, $k_b T_L$, $k_b T_X$) as (170, 40, 37), (300, 50, 38), (340, 200, 40), (380, 325, 50), (410, 470, 60) in meV. Expected values of n_Γ, n_X, T_Γ and T_X against x-coordinate along the channel are shown in Supplementary Figs. 8 a and b. For the estimate, we note that, in the experiment, (i) $E \approx$ 40 kV/cm in the channel, (ii) the contribution of L valleys is ignored and (iii) hot LO-phonon distribution raises T_Γ, T_X and n_X, compared to the simulation of Ref. 35. We mention that T_X can be defined only in the region of non-zero n_X. The average electron temperature, $\langle T_e \rangle = (n_\Gamma T_\Gamma + n_X T_X)/n$, is derived from n_Γ, n_X , T_Γ and T_X, and is shown together in Supplementary Fig. 8 b (also plotted in Fig. 3c). The curve exhibits a double-peak structure as experimentally observed.

Expected profile of LO-phonon temperature $T_{LO}(x)$ is shown together with $T_X(x)$ in Supplementary Fig. 8 c. We note that $T_{LO}(x)$ is larger than $T_X(x)$ but very close to $T_X(x)$ in the channel because X-valley electrons play a decisive role in reabsorbing LO-phonons due to their density of states far larger than that of Γ valley, $D_X(\varepsilon+\Delta\varepsilon_{\Gamma X})/D_\Gamma(\varepsilon) = (m^X{}_d/m^\Gamma{}_d)^{3/2} \approx 66$. Outside the channel exit, T_{LO} gets substantially lower than T_X and falls smoothly to 300 K. As the entrance is approached on the entrance side, T_{LO} is elevated prior to the increase of n_X.

Knowing the values of n_Γ, n_X, T_Γ, T_X and T_{LO} we can derive $P_{LO}(x)$ through Eq. (4) if the distribution function, $f_i(\varepsilon) = 1/[\exp\{(\varepsilon - \mu_i)/k_B T_i\} + 1]$, is determined for each valley (i = Γ or X).

This can be done by deriving the position dependent electrochemical potential, $\mu_i(x)$, from n_i (x) and $T_i(x)$ at each position x for each valley i, by imposing the equation

$$n_i\,(x) = \int_0^{\infty} f_i(\varepsilon) D_i(\varepsilon) d\varepsilon.$$

The derived curve of P_{LO} is shown in Supplementary Fig. 8 d and in Fig. 3d.

The probability of the electrons passing through the channel without dissipation can be estimated from the profile of P_{LO} in Supplementary Fig. 8 d by defining it to be the ratio of the value of P_{LO} integrated outside the channel exit to the total integrated value, p_{exit}/p_{total}. We obtain p_{exit}/p_{total} ≈ 0.93, where $p_{total} = p_{exit} + p_{channel}$ with $p_{exit} \propto S_{exit} \langle P_{LO} \rangle_{exit}$ and the fractional power dissipation in the channel $p_{channel} \propto S_{channel} \langle P_{LO} \rangle_{channel}$, where $S_{exit} \approx \pi (0.6)^2 \mu m^2$ is an approximate area of substantial power dissipation outside the exit, $S_{channe} \approx (0.4 \times 0.87)\ \mu m^2$ the channel area, and $\langle P_{LO} \rangle_{exit} / \langle P_{LO} \rangle_{channel} \approx 2.2/0.55$ is the ratio between the average power density dissipations in the channel exit and in the channel determined from Supplementary Fig. 8 d.

The suppression ratio of energy loss γ_{supp} is defined, similarly to Eq. S3, by the ratio of P_{LO} to the fictitious loss rate P_0 realized without the hot-phonon bottleneck effect; viz.,

$$\gamma_{supp} = P_{LO}(T_{LO})/P_0,$$

where P_0 is evaluated by integrating $P_{LO}(x)$ over x with all the variables unchanged in Eq. (4) except that T_{LO} is replaced with $T_{base} = 300.8$ K. Supplementary Figure 9 shows that $\gamma_{supp} = P_{LO}/P_0$ drops to about 1% in the channel, demonstrating remarkable hot-phonon bottleneck effect. (It is reasonable that the value $\gamma_{supp} \sim 1\%$ derived here is larger than $\gamma_{supp} = 0.37\%$ obtained from the simplified single-carrier model in section *"VI. Self-consistent determination of T_{LO} in a simplified model"* in Supplementary Information.)

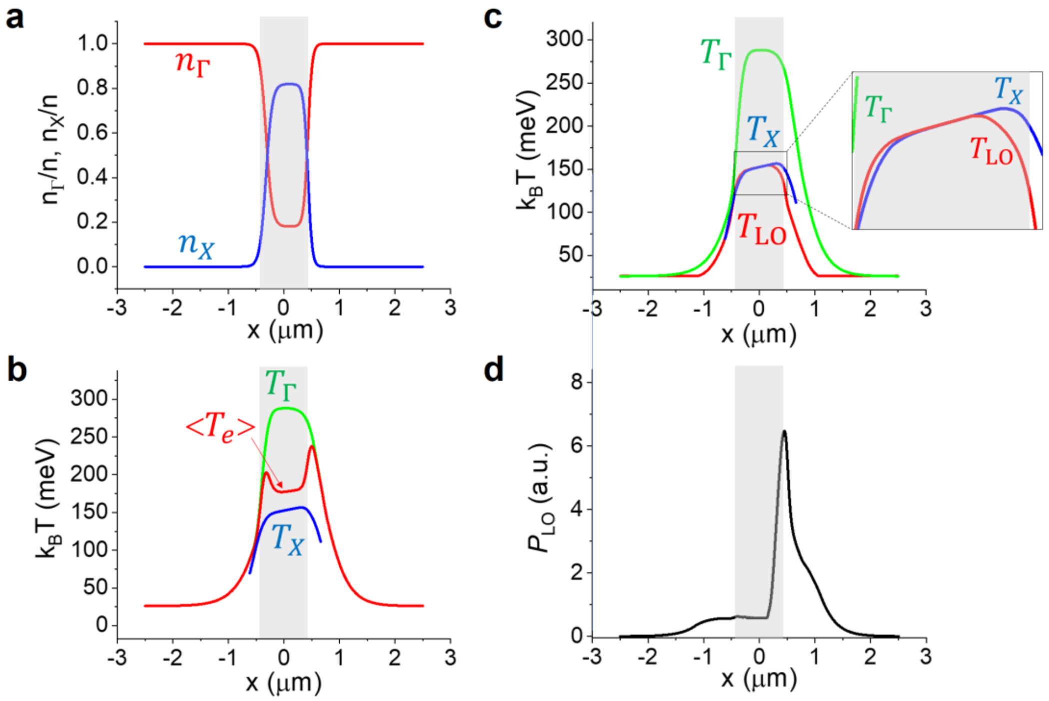

Supplementary Figure 8. Expected values of important physical quantities along the channel of the device of Figs. 1 & 3 and Supplementary Figs. 3 & 4 with V_b = 8.0 V. Electron current flows from the left (-x) to the right (+x). Shadings mark the channel region. (a) n_Γ, n_X , (b) T_Γ, T_X, $<T_e>$ = $(n_\Gamma T_\Gamma + n_X T_X)/n$, and (c) T_{LO} is compared with T_Γ and T_X. (d) P_{LO} is the net energy loss due to LO-phonon scattering. In (c), $T_X(x)$ is replotted from (b) for comparison with T_{LO}.

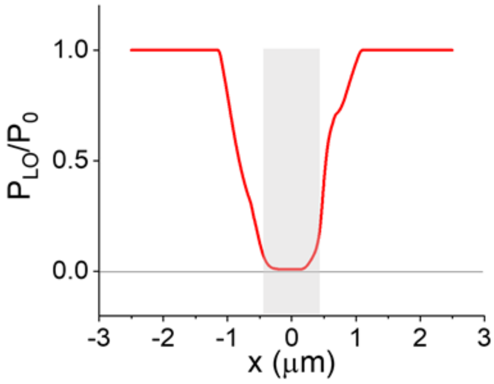

Supplementary Figure 9. Suppression ratio of energy loss due to the hot-phonon bottleneck effect for the device of Figs. 1 & 3 and Supplementary Figs. 3 & 4 with V_b = 8.0 V.

Supplementary Note 9 Broadening of T_L distribution

Energy flux P_{LO} (x) fed to the lattice system eventually relaxes to the heat, which, in turn, spreads via lattice heat conduction. If heat is steadily fed to a point $\boldsymbol{r} = 0$ on a surface of a half-infinite uniform material of temperature T_L, heat flux $\boldsymbol{h}$ is generated radially propagating outwards from the origin $\boldsymbol{r} = 0$, heating the surrounding region to $T_L + \Delta T_L(r)$. The heat flux is driven by temperature gradient, $\boldsymbol{h} \propto -\nabla(\Delta T_L)$, while the amplitude of $\boldsymbol{h}$ decays as $1/r^2$. It follows that $\partial(\Delta T_L)/\partial r \propto -1/r^2$ and

$$\Delta T_L(r) \propto 1/r.$$

This relation is supported by the experimental observation; that is, the profile of $\Delta T_L(y)$ along line 3 (crossing the hot spot) in Supplementary Fig. 4 b is approximately fitted by the curve $\Delta T_L(y) \propto 1/[1 + (|y|/D)]$ with D= 600 nm the broadening parameter.

We derive the $T_L(x)$ curve in Fig. 3d by considering $1/[1 + (|x-x_i|/D)]$ with D= 600 nm for the spread of the heat originating at $x=x_i$ and by superposing the contribution from the entire channel region by weighting with $P_{LO}(x)$.

Supplementary References

1. Komiyama, S. Single-photon detectors in the terahertz range. *IEEE J. Sel. Top. Quantum Electron*. **17**, 54-66 (2011).
2. Ueda, T. & Komiyama, S. Charge-sensitive Infrared Phototransisotrs: Single-photon Detectors in the Long-Wavelength Infrared. *Sensors & Transducers Journal.* **10**, Special Issue, 60-70 (2011).
3. Kajihara, Y., Kosaka, K. & Komiyama, S. A sensitive near-field microscope for thermal radiation. *Rev. Sci. Instrum.* **81**, 033706 (2010).
4. Komiyama, S. et al. Near-field Nanoscopy of Thermal Evanescent Waves on Metals. *arXiv:* 1601.00368.
5. Kajihara,Y., Kosaka, K. & Komiyama, S. Thermally excited near-field radiation and far-field interference. *Opt. Express* **19**, 7695-7704 (2011).
6. Weng, Q. et al. Near-field radiative nanothermal imaging of nonuniform Joule heating in narrow metal wires. *Nano Lett.* **18**, 4220-4225 (2018).
7. Weng, Q. et al. Imaging of nonlocal hot-electron energy dissipation via shot noise. *Science* **360**, 775-778 (2018).
8. Komiyama, S. Perspective: Nanoscopy of charge kinetics via terahertz fluctuation. *J. Appl. Phys*. **125**, 010901 (2019).
9. Hirakawa, K. et al., Blackbody radiation from hot two-dimensional electrons in $Al_xGa_{1-x}As$/GaAs heterojunctions. *Phys. Rev.* B **47**, 16651(1993).
10. Maeng, I. et al., Strong emission of THz radiation from GaAs microstructures on Si. *AIP Advances* **8**, 125027 (2018).
11. Ikushima, K. et al., Visualization of quantum Hall edge channels through imaging of terahertz emission. *Phys. Rev.* B **76**, 165323 (2007).
12. Lin, K. -T., Komiyama, S. & Kajihara, Y. Tip size dependence of passive near-field microscopy. *Opt. Lett.* **41**, 484 (2016).
13. Weng, Q. et al. Comparison of active and passive methods for the infrared scanning near-field microscopy. *Appl. Phys. Lett.* **114**, 153101 (2019).
14. Joulain, K. et al. Surface electromagnetic waves thermally excited: Radiative heat transfer, coherence properties and Casimir forces revisited in the near field. *Surf. Sci. Rep.* **57**, 59–112 (2005).
15. Biehs, S. -A., Reddig, D. & Holthaus, M. Thermal radiation and near-field energy density of thin metallic films. *Eur. Phys. J.* B **55**, 237-251 (2007).
16. Joulain, K. et al. Definition and measurement of the local density of electromagnetic states close to an interface, *Phys. Rev. B* **68**, 245405 (2003).
17. Shi, L., et al., Thermal Transport Mechanisms at Nanoscale Point Contacts *J. Heat Transfer* **124**, 329 (2002).
18. Menges, F. et al. Temperature mapping of operating nanoscale devices by scanning probe thermometry. *Nat. Commun*. **7**, 10874 (2016).
19. Zhang, Y. et al. A review on principles and applications of scanning thermal microscopy (SThM). *Adv. Funct. Mater*. **30**, 1900892 (2019).
20. Aigouy, L. et al. Imaging current paths in complex conductors by scanning fluorescence microscopy. *Appl. Phys. Lett.* **101**, 123113 (2012).

21. Goodwill, J. M. et al. Spontaneous current constriction in threshold switching devices. *Nat. Commun.* **10**, 1628 (2019).
22. Halbertal, H. et al. Nanoscale thermal imaging of dissipation in quantum systems. *Nature* **539**, 407-410 (2016).
23. Marguerite, A. et al. Imaging work and dissipation in the quantum Hall state in graphene. *Nature* **575**, 628-633 (2019).
24. Mecklenburg, M. et al. Nanoscale temperature mapping in operating electronic devices. *Science* **347**, 629 (2015).
25. Ward, D. R. et al. Vibrational and electronic heating in nanoscale junctions. *Nature Nanotechnol.* **6**, 33 (2011).
26. Kloppstech, K. et al. Dancing the tight rope on a nanoscale –Calibrating a heat flux sensor of a scanning thermal microscope. *Rev. Sci. Instrum.* **86**, 114902 (2016).
27. Rosenwaks, Y. et al. Hot-carrier cooling in GaAs: Quantum wells versus bulk. *Phys. Rev. B* **48**, 14675 (1993).
28. Maloney, T. J. & Frey, J. Transient and steady-state electron transport properties of GaAs and InP. *J. Appl. Phys.* **48**, 781 (1977).
29. Jyegal, J. Velocity overshoot decay mechanisms in compound semiconductor field-effect transistors with a submicron characteristic length. *AIP Advances* **5**, 067118 (2015).
30. Hu, X. et al. Enhanced Peltier effect in wrinkled Graphene constriction by nano-bubble engineering. *Small* **16**, 1907170 (2020).
31. Homm, G. et al. Seebeck coefficients of *n*-type (Ga,In)(N,As),(B,Ga,In)As, and GaAs. *Appl. Phys. Lett.* **93**, 042107 (2008).
32. Aninkevičius, V. et al. Γ-X intervalley-scattering time constant for GaAs estimated from hot electron noise spectroscopy data. *Phys. Rev. B* **53**, 6893–6895 (1996).
33. Blakemore, J. S. Semiconducting and other major properties of gallium arsenide. *J. Appl. Phys.* **53**, R123–R181 (1982).
34. Majumdar, A., Fushinobu, K., and Hijikata, K. Effect of gate voltage on hot-electron and hot phonon interaction and transport in a submicrometer transistor. *J. Appl. Phys.* **77**, 6686 (1995).
35. Požela, J. & Reklaitis, A. Electron transport properties in GaAs at high electric fields. *Solid-State Electron.* **23**, 927–933 (1980).
